# Compositional and drug-resistance profiling of pathogens in patients with severe acute pancreatitis: a retrospective study

**DOI:** 10.1186/s12876-020-01563-x

**Published:** 2020-12-01

**Authors:** Ning Fan, Yong Hu, Hong Shen, Shengjie Liu, Guang Zhao, Lanju Sun, Chunyan Li, Xin Zhao, Yanning Li, Jianhua Wang, Yunfeng Cui

**Affiliations:** 1 Department of Surgery, Beichen Chinese Medicine Hospital, Tianjin University of Traditional Chinese Medicine, Tianjin, China; 2 Zhongnan Hospital of Wuhan University, No. 167, Donghu Road, Wuchang District, Wuhan, 430000 China; 3 Department of Surgery, Characteristic Medical Center of the Chinese People’s Armed Police Force, Tianjin, China; 4 Tianjin University of Traditional Chinese Medicine, Tianjin, China; 5 Tianjin Nankai Hospital, Nankai Clinical School of Medicine, Tianjin Medical University, Tianjin, China; 6 Department of Gastroenterology, Affiliated Hospital of Panzhihua University, Nanchong, China; 7 Department of Breast Surgery,Affiliated Cancer Hospital of Zhengzhou University, Henan Cancer Hospital, Zhengzhou, China; 8 Department of Surgery, Tianjin Nankai Hospital, Nankai Clinical School of Medicine, Tianjin Medical University, 122 Sanwei Road Nankai District, Tianjin, China

**Keywords:** Severe acute pancreatitis, Multi-drug resistant bacteria, Bacteria spectrum, Antibiotic resistance, Risk factors

## Abstract

**Background:**

Infection is one of the important causes of death in patients with severe acute pancreatitis (SAP), but the bacterial spectrum and antibiotic resistance are constantly changing. Making good use of antibiotics and controlling multi-drug-resistant (MDR) bacterial infections are of vital importance in improving the cure rate of SAP. We conducted a retrospective study in the hope of providing references for antibiotic selection and control of drug-resistant bacteria.

**Methods:**

Retrospective analysis was performed on the data of patients hospitalized in our hospital due to acute pancreatitis (AP) in the past 5 years. General data were classified and statistically analyzed. Subsequently, the bacterial spectrum characteristics and the data related to drug-resistant bacterial infection of 569 AP patients were analyzed. Finally, unconditional logistic regression analysis was conducted to analyze the risk factors of MDR infection.

**Results:**

A total of 398 patients were enrolled in this study and the hospitalization data and associated results were analyzed. A total of 461 strains of pathogenic bacteria were detected, including 223 (48.4%) gram-negative bacterial strains, 190 (41.2%) gram-positive bacterial strains and 48 (10.4%) fungal strains. The detection rates of resistance in gram-negative and gram-positive bacterial strains were 48.0% (107/223) and 25.3% (48/190), respectively. There were significant differences between the MDR group and the non-MDR group for the factors of precautionary antibiotic use, kinds of antibiotics used, receipt of carbapenem, tracheal intubation, hemofiltration and number of hospitalization days in the intensive care unit. Unconditional logistic regression revealed 2 risk factors for MDR bacterial infection.

**Conclusions:**

Our results illustrate that gram-negative bacteria were the most common pathogens in SAP infection, and the proportion of gram-positive bacteria increased notably. The rate of antibiotic resistance was higher than previously reported. Unconditional logistic regression analysis showed that using more types of antibiotics and the number of hospitalization days in the ICU were the risk factors associated with MDR bacterial infection.

## Introduction

Acute pancreatitis (AP) is an inflammatory injury with pancreatic edema, hemorrhage and necrosis caused by the self-digestion of pancreatic tissue. Clinical features include acute upper abdominal pain and the elevation of amylase or lipase. AP is classified as mild acute pancreatitis (MAP), moderate to severe acute pancreatitis (MSAP) and severe acute pancreatitis (SAP). SAP is a critical condition with poor prognosis in the clinic. The mortality rate may be up to 30% [1] due to local and systemic complications, including systemic inflammatory response syndrome (SIRS), acute respiratory distress syndrome (ARDS) or multiple organ dysfunction syndrome (MODS) in the early stage [2−4]. Subsequently, infectious pancreatic necrosis (IPN) will appear in approximately 40 to 70% of patients in the second stage [5, 6], and the mortality rate can be as high as 32 to 50% [7–8] Currently, the treatment of IPN has evolved from open surgery to comprehensive treatment based on minimally invasive techniques, such as endoscopic treatments, percutaneous drainage and minimally invasive necrotic tissue removal [9−11].

Antibiotics are used for almost the entire treatment process [12]. This is because pancreatic and peripancreatic infectious necrosis is mainly caused by intestinal bacterial translocation [13, 14]. However, existing control methods cannot effectively prevent this process, a series of complex infections, such as MDR bacterial infections and fungal infections, often occur in the course of disease development. As such, it is necessary to actively seek prevention and treatment strategies; early use of antibacterial drugs to prevent pancreatic infection is a common method. Because some antibacterial drugs cannot effectively act on the pancreas in the case of systemic drug delivery, there is no effective antibacterial drug that can penetrate necrotic tissue without a blood supply, which increases the difficulty of antibiotic treatment [15]. However, imipenem, clindamycin, piperacillin, fluoroquinolone and metronidazole have sufficient tissue penetration and bactericidal properties for infectious pancreatic necrosis and have certain advantages in preventing and treating IPN [16]. There is still controversy about the prophylactic use of antibiotics to prevent infection [17, 18]. How to identify pancreatic infections early, how to choose antibacterial drugs and how to time treatments are still major problems to be solved in the clinic.

The main purpose of this study includes two aspects. First, analyzing the characteristics of the bacterial spectrum and the changes in antibiotic resistance in AP patients is helpful to guide the preventive and empirical use of antibiotics. Second, the analysis of the risk factors for MDR bacterial infections can help doctors avoid particular treatments when controlling infections and delay or reduce MDR bacterial infections as much as possible.

## Methods

### General information

The data of MAP, MSAP and SAP patients hospitalized in our hospital were collected respectively, according to their diagnoses at the time of discharge. The time range of MAP and MSAP groups was from January 1, 2019 to December 31, 2019. The SAP group collected data over the past five years from January 1, 2015 to December 31, 2019. The patients in SAP group were divided into MDR group and Non-MDR group according to whether they had MDR infection. All patients must meet inclusion and exclusion criteria.

### Inclusion and exclusion criteria

The inclusion criteria were as follows: (1) the patients met the AP diagnostic criteria proposed by the Atlanta consensus meeting [19], and (2) the results of the bacterial culture confirmed the pathogen diagnosis for infection. The exclusion criteria were as follows: (1) patients experiencing pregnancy-associated pancreatitis (2) the presence of a malignant tumor, (3) long-term use of immunosuppressive agents or patients with immune deficiency diseases, and (4) patients with incomplete hospitalization data.

### Data collection instructions

Bacterial culture data and drug sensitivity test data were collected, including abdominal drainage fluid, sputum, blood, bile, deep venous catheter, and urine. The pathogens from the same patient and specimens were not counted repeatedly.

The date collection time points were as follows: at admission, 2 weeks after admission, 1 month after admission, and when the patient's clinical condition showed a major turning point. These changes include: (1) the body temperature was above 38 °C or below 36 °C; (2) the patient experienced tachycardia, persistent hypotension (systolic blood pressure below 90 mmHg) or shortness of breath; (3) the patient experienced chills; (4) white blood cell counts increased or were extremely low (white blood cell count (WBC): 18,000 cells /mm^3^ or WBC < 4000 cell/mm^3^); (5) platelet counts were < 150,000 cell/mm^3^, (6) the patient experienced an unexplained elevated CRP in the immunosuppressed state; (7) creatinine levels were > 2.0 mg/dL; or (8) other suspected conditions worsened [20−22].

Risk factors were selected according to the factors that may cause MDR infection as reported in previous literature and clinically invasive procedures [23−26].

### Definitions

MDR bacteria were defined by the following criteria [27]: (1) third-generation cephalosporin-resistant, (2) β-lactam-resistant Enterobacteriaceae (e.g., *Escherichia coli*, *Klebsiella pneumonia*, and *Serratia marcescens*), (3) MDR gram-negative rods defined as other gram-negative rods not susceptible to at least one agent in three or more antimicrobial categories (e.g., *Acinetobacter baumannii*, and *Pseudomonas aeruginosa*), (4) methicillin-resistant *Staphylococcus aureus* (MRSA), (5) methicillin-resistant, coagulase-negative staphylococci (MRCNS), and (6) vancomycin-resistant *Enterococcus* species (VRE).

### Strain treatment

The specimens were cultured using a French BioMerieux BacT/ALERT 3D automatic blood training instrument and a CO_2_ incubator. A VITEK 2 compact automatic microbiological analyzer was used to identify the positive specimens for drug susceptibility tests. The susceptibility test was based on a breakpoint set by the American Association of Clinical Laboratory Standards (CLSI) in 2015 to determine drug resistance [28]. The quality control strains included *Escherichia coli* ATCC25922, *Enterobacter cloacae* ATCC700323, *Staphylococcus aureus* ATCC29213 and *Streptococcus pneumoniae* ATCC49619.

### Statistical analysis

WHONET V.5.6 for Windows (WHO Collaborating Center, Boston) was used to collect the data and analyze the pathogens for drug resistance. SPSS V.22.0 for Windows (SPSS Inc., Chicago, IL) was used for statistical analysis, and enumeration data were checked by the chi-square test according to whether the measurement data were normally distributed. A t-test or rank sum test was performed. Fisher's exact test was used for the dichotomous contingency table, according to variable types. A univariate analysis was included in the unconditional logistic regression analysis model to calculate odds ratios (ORs) along with 95% confidence intervals (CIs) to assess the strength of any association, and a 2-sided *P* < 0.05 was considered statistically significant.

## Results

### Clinical data comparison

**Fig. 1:**
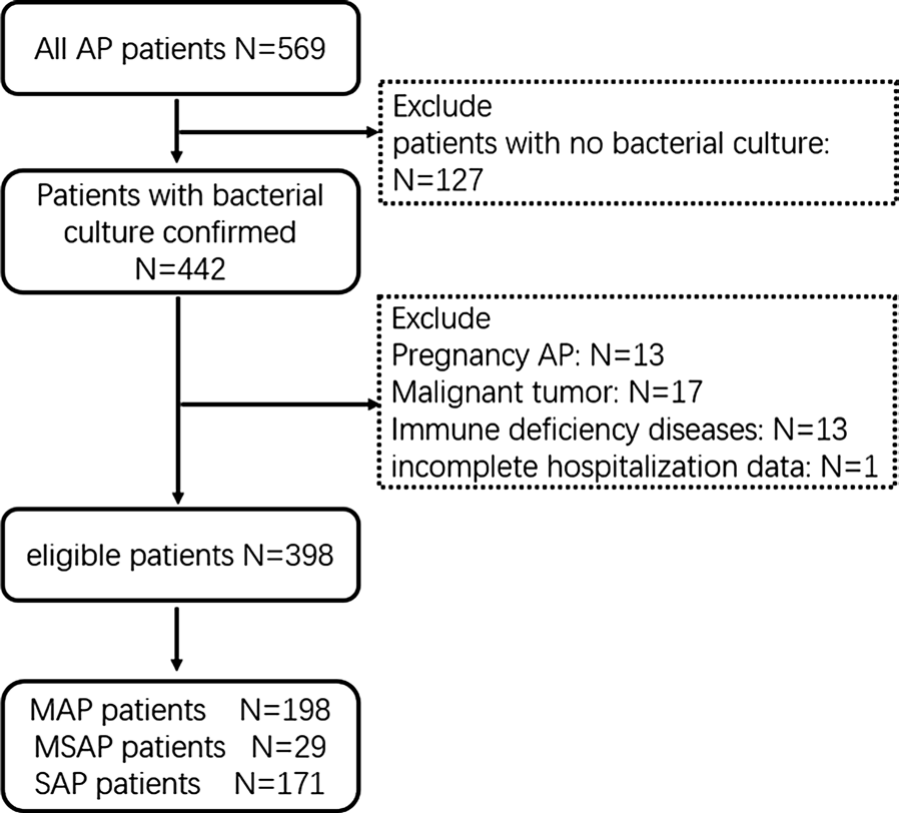
Flow diagram of the patients with AP in the study

A total of 569 AP patients were retrieved, 127 were excluded owing to the lack of bacterial culture, and 398 were eligible according to the exclusion criteria. There were 198 patients in MAP group, 29 in MSAP group and 171 in SAP group. A detailed study flow-diagram is shown in Fig. 1.

Statistical analysis showed that the total hospitalization days was significantly different among the MAP group, MSAP group and SAP group (*P* = 0.000). The death rate of the SAP group was significantly higher than that of the MAP group and MSAP group (*P* = 0.000). This is consistent with the severity of the disease (Table 1).

**Table 1:** Clinical data from the MAP, MSAP and SAP group

Items	MAP	MSAP	SAP
Eligible number	198	29	171
Male	111	18	97
Femal	87	11	74
Age	54.5 ± 16.7	53.8 ± 15.4	52.4 ± 16.3
Etiology			
Chololithiasis	129	15	83
Hyperlipidaemia	44	8	36
Alcohol	15	6	28
Others	10	0	24
Total hospitalization days	8.0 ± 3.6	24.0 ± 14.4	54 ± 43.6
Death rate in hospital	0	0	22

Of the 171 patients with SAP, 97 were male and 74 were female, with an average age of 52.4 ± 16.3 years old. The patients were divided into an MDR group (81 cases, 47.4%) and a non-MDR group (90 cases, 52.6%) according to whether MDR bacterial infection was present. There were no significant differences in sex, age, cause of disease, severity of SAP, number of fungal infections and total hospitalization days between the two groups (Table 2).

**Table 2:** Clinical data from the MDR group and non-MDR group

Characteristic	MDR (n = 81)	non-MDR (n = 90)	*P*-value
Gender			
Male	44 (54.3%)	53 (58.9%)	0.547
Female	37 (45.7%)	37 (41.1%)	
Age	52.4 ± 16.9	52.3 ± 15.7	0.972
Cause			
Biliary	38 (46.9%)	45 (50.0%)	0.678
Hyperlipidemia	19 (23.5%)	17 (18.9%)	0.464
Alcohol	13 (16.1%)	15 (16.7%)	0.913
Others^a^	11 (13.6%)	13 (14.4%)	0.683
BISAP score	3.0 ± 1.0	2.6 ± 1.1	0.206
CTSI score	6.6 ± 1.4	6 ± 1.5	0.104
APACHE II score	15.6 ± 8.6	14.1 ± 6.5	0.204
MODS	45 (55.6%)	38 (42.2%)	0.082
SIRS^b^	81 (100.0%)	90 (100.0%)	–
Fungal infection	28 (34.6%)	21(23.3%)	0.105
Total hospitalization days	45.3	36.1	0.062
Death rate in hospital	15 (18.5%)	7 (7.8%)	0.036

^a^SAP with unknown etiology

^b^All patients had SIRS complications, and we were unable to perform independent statistical analyses

### Distribution of pathogens

A total of 654 strains of pathogenic bacteria were detected, including 348 strains of gram-negative bacteria, 259 strains of gram-positive bacteria and 48 strains of fungi (Fig. 1).

**Fig. 2:**
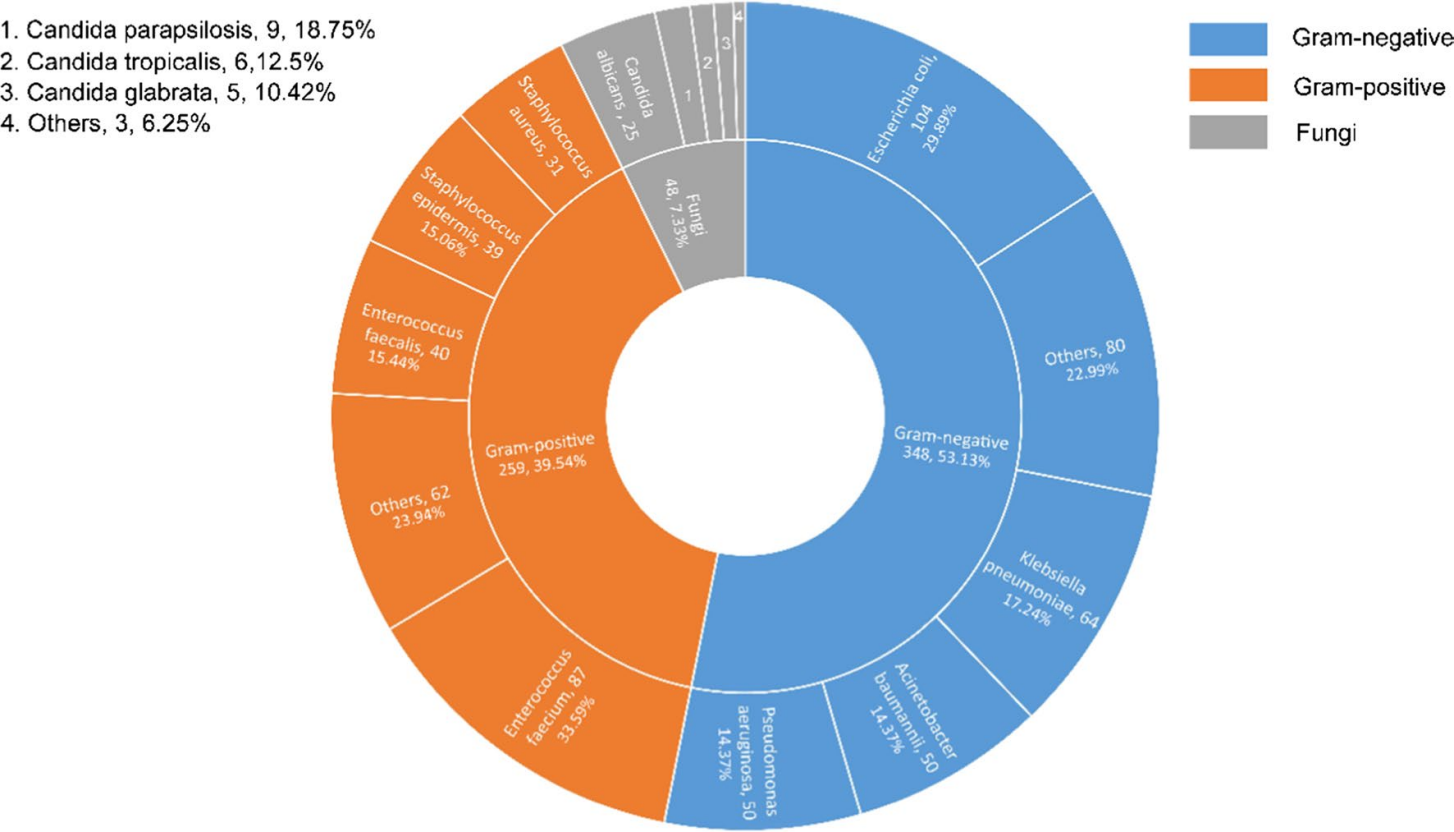
Strains composition diagram

In the SAP group, 461 pathogenic strains were detected, among which 223 strains were gram-negative and 190 strains were gram-positive. *Escherichia coli* was the dominant bacterium in gram-negative bacteria, while enterococcus faecium was the dominant bacteria group in gram-positive bacteria. All the fungi were detected in the SAP group . The source and strain quantities of pathogens are shown in Fig. 2.

### Main bacterial composition and MDR distribution in SAP group

**Fig. 3:**
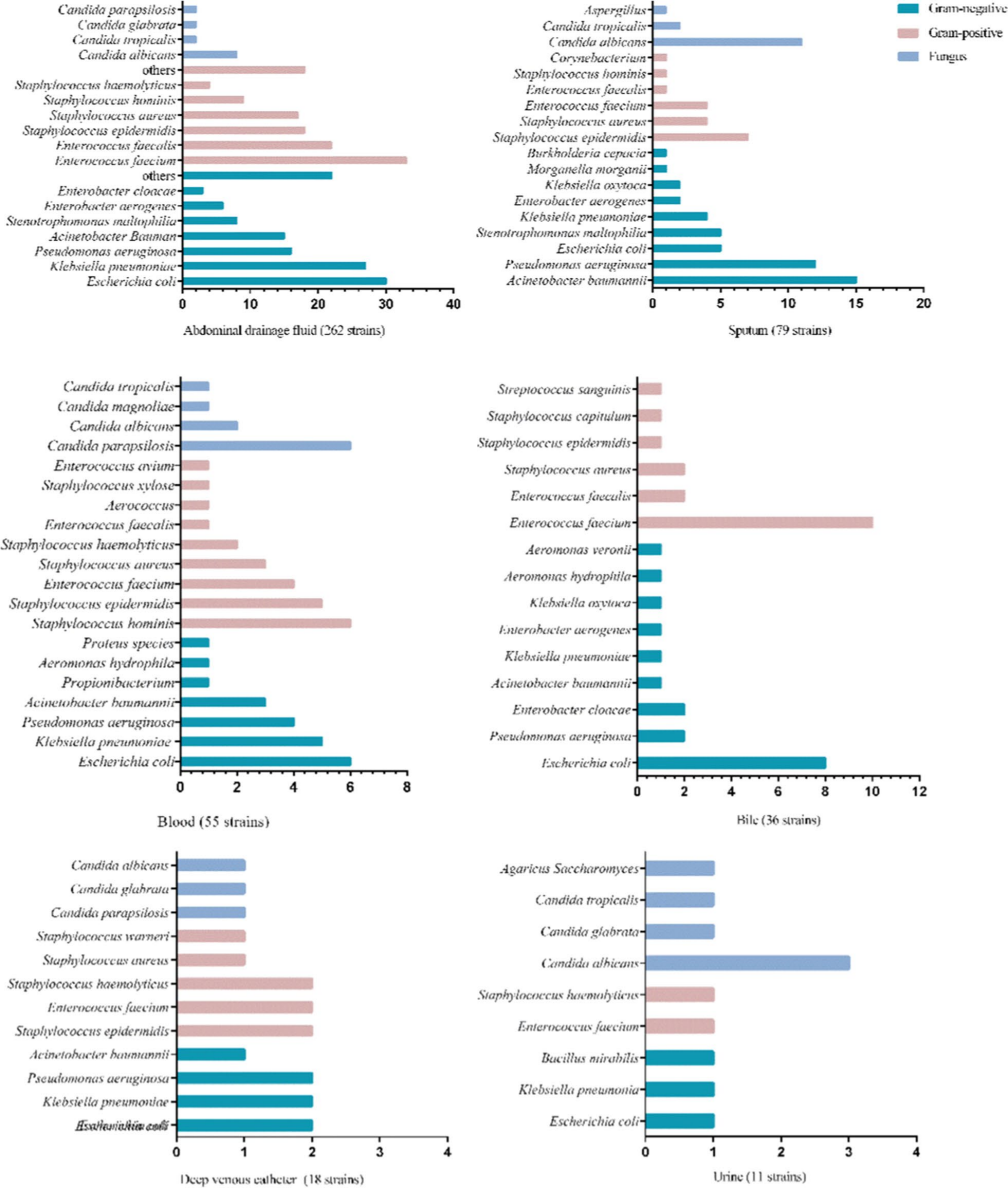
Distribution of pathogen strains in the SAP group

**Fig. 4:**
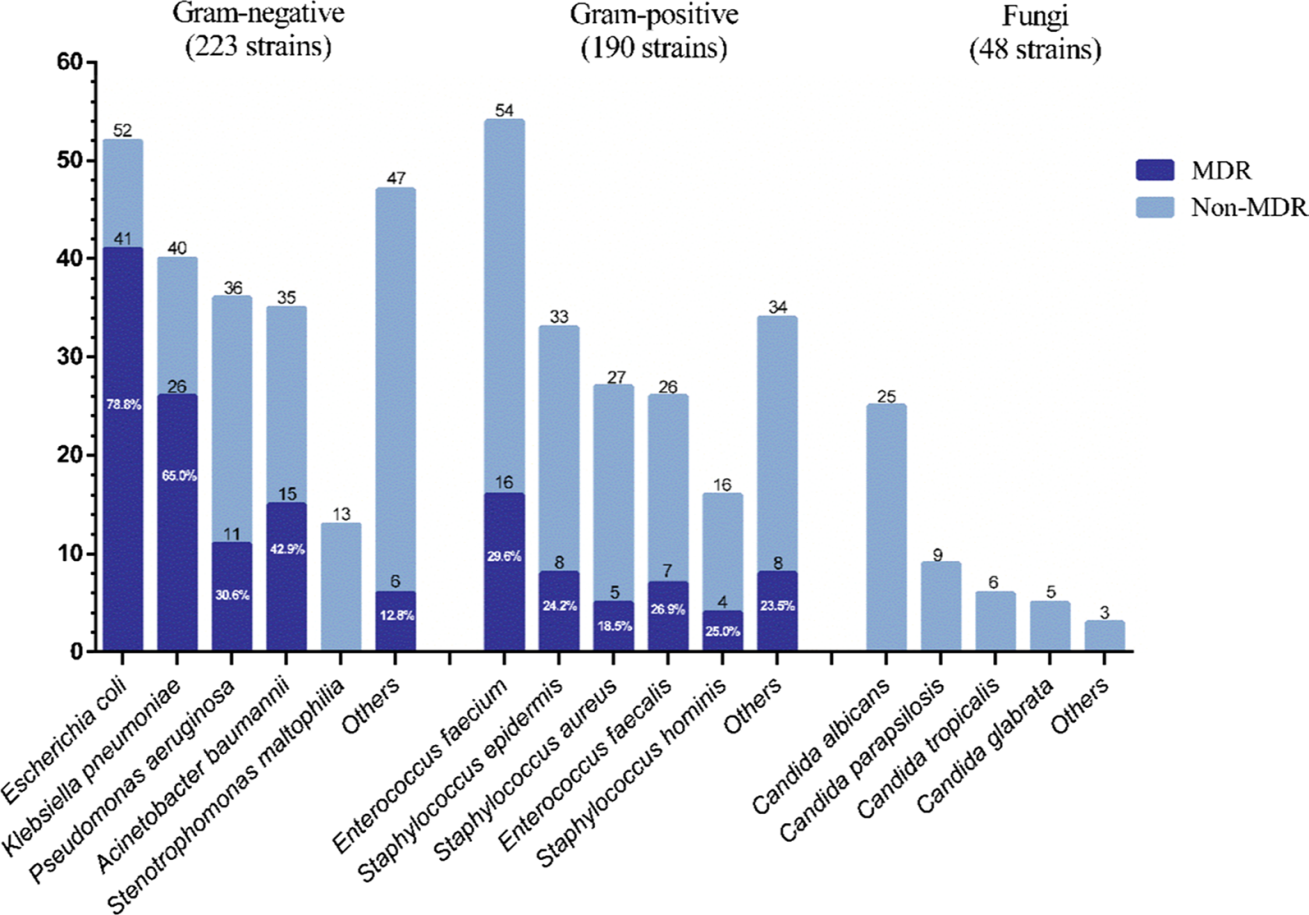
Main bacterial composition and MDR distribution in SAP group

A total of 223 strains of gram-negative bacteria were detected in the SAP group, including 99 strains of MDR and 8 strains of extensively drug-resistant bacteria (XDR) *Pseudomonas aeruginosa*. For gram-positive bacteria, the detection rate of MDR bacteria was 25.3% (48/190). There were 48 strains of fungus and no resistant strains (Figs. 3, 4).

### Antibiotic resistance analysis in SAP group

#### Gram-negative

**Fig. 5:**
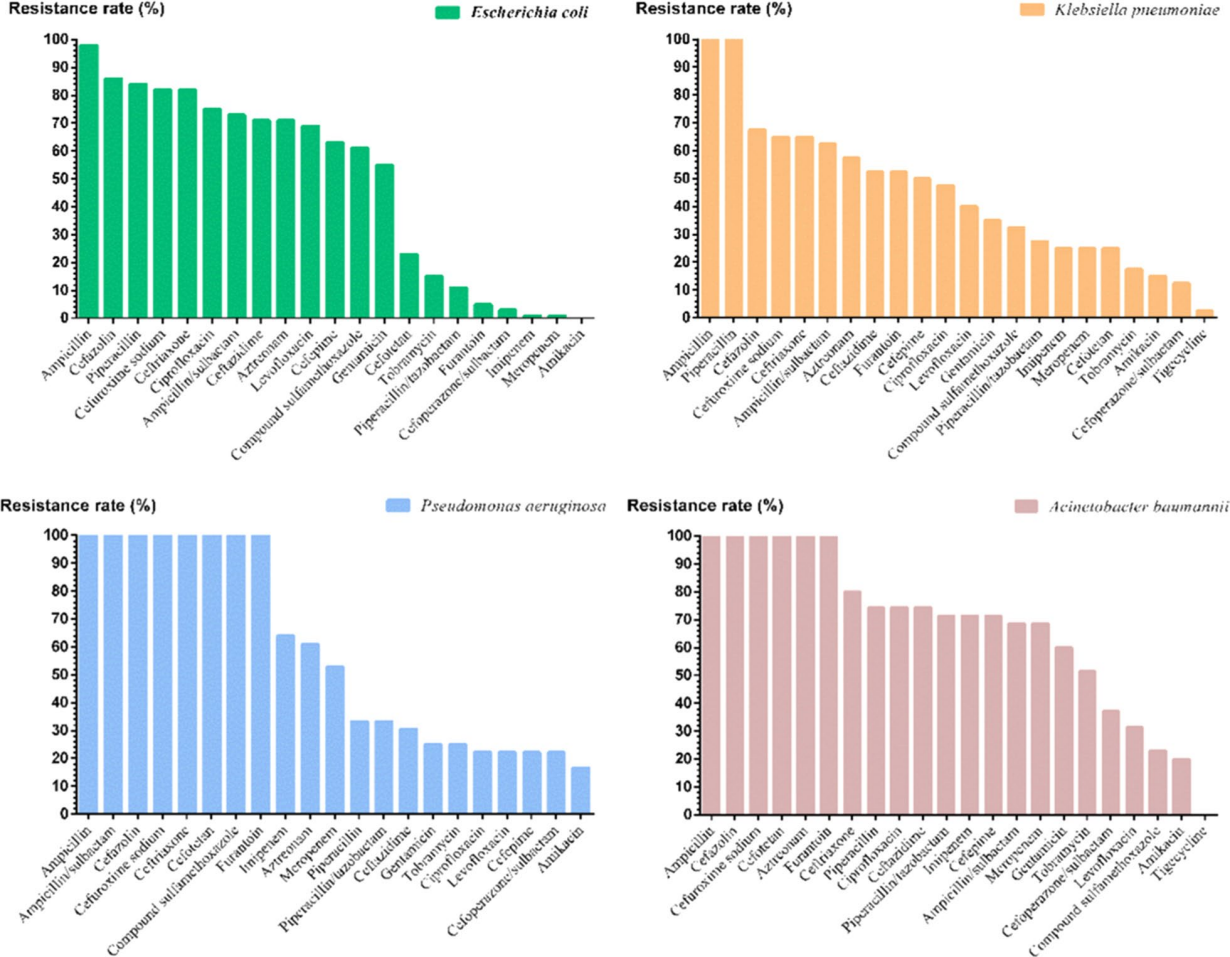
The main gram-negative bacteria resistance rate

The detection rates of extended-spectrum beta-lactamases (ESBL) in *Escherichia coli* and *Klebsiella pneumoniae* were 82.7% (43/52) and 65% (26/40), respectively. *Escherichia coli* and *Klebsiella pneumoniae* were sensitive to carbapenems, but the resistance rates of *Pseudomonas aeruginosa* and *Acinetobacter baumannii* to carbapenems were all over 50%. The main bacterial strains were sensitive to piperacillin/tazobactam, except for *Acinetobacter baumannii*. The main gram-negative bacteria were sensitive to cefoperazone/sulbactam. A strain of tigecycline-resistant *Klebsiella pneumoniae* was detected (Fig. 5).

#### Gram-positive

**Fig. 6:**
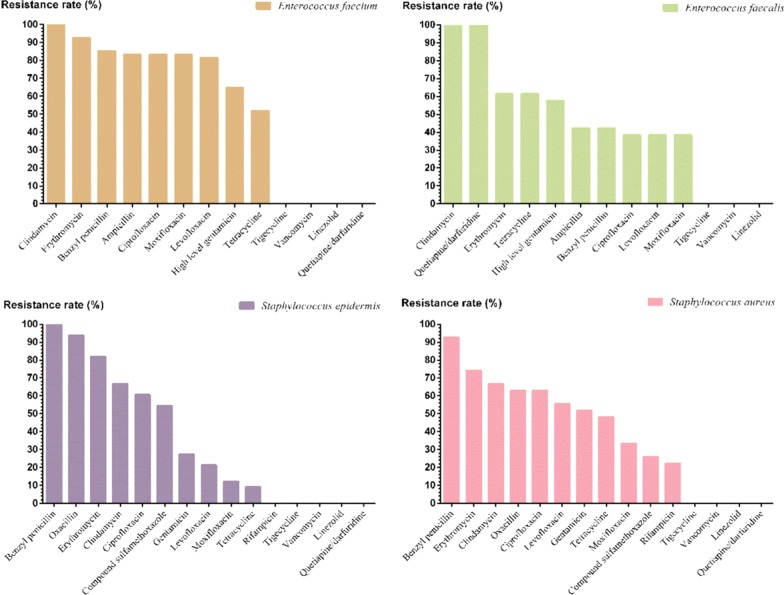
Main gram-positive bacteria resistance rate

The detection rate of MRSA was 10.5% (20/190) and that of MRCNS was 2.1% (4/190). Among the enterococci, the resistance rates of *Enterococcus faecium* to benzyl penicillin and ampicillin were 85.2% and 83.3%, respectively, while the resistance rates of *Enterococcus faecalis* were both 42.3%. The resistance rates of *Enterococcus faecium* and *Enterococcus faecalis* to high concentration gentamicin combined with ampicillin were 64.8% and 57.7%, respectively. There were no strains resistant to vancomycin, temozolomide or linezolid (Fig. 6).

### Comparison of MDR infection factors

A total of 12 risk factors for MDR bacterial infection in the MDR and non-MDR groups were compared and included the following: precautionary antibiotic use, kinds of antibiotics used, use of carbapenem antibiotics, use of aminoglycoside antibiotics, average number of days of antibiotic use, endoscopic operation, intraperitoneal catheterization, venipuncture, preservation of the catheter, tracheal intubation, hemofiltration and number of hospitalization days in the ICU. Univariate analysis revealed 6 statistically significant infection factors, namely, precautionary antibiotic use (*P* = 0.030), kinds of antibiotic used (*P* = 0.005), use of carbapenem antibiotics (*P* = 0.009), tracheal intubation (*P* = 0.029), hemofiltration (*P* = 0.047) and number of hospitalization days in the ICU (*P* = 0.018) (Table 3).

**Table 3:** comparison of infection factors between the MDR group and non-MDR group

Infection factors	MDR (n = 81)	non-MDR (n = 90)	*P*-value
Precautionary antibiotics	44 (54.3%)	34 (37.8%)	0.030
Kinds of antibiotics	3	2	0.005
Carbapenems	58 (71.6%)	47 (52.2%)	0.009
Aminoglycosides	8 (9.9%)	7 (7.8%)	0.628
Antibiotic days^a^	34.1	27.0	–
Endoscopic operation^b^	10 (12.3%)	10 (11.1%)	0.802
Intraperitoneal catheterization^c^^§^	81 (100.0%)	90 (100.0%)	–
Venipuncture^d^	81(100.0%)	90 (100.0%)	–
Catheter preservation^d^	81 (100.0%)	90 (100.0%)	–
Tracheal intubation	26 (32.1%)	16 (17.8%)	0.029
Hemofiltration	9 (11.1%)	3 (3.3%)	0.047
Hospitalization days in the ICU	13	4	0.018

^a^Because of the social and human factors, the credibility of the data was low, so it was excluded.

^b^Including ERCP, ENBD, endoscopic ultrasonography-guided puncture drainage, etc.

^c^Intraperitoneal catheterization included intraoperative abdominal catheterization and B ultrasound/CT guided abdominal puncture drainage

^d^All patients were included and were unable to perform independent statistical analyses

### Risk factors

MDR bacterial infection was the dependent variable, and the independent variables were precautionary antibiotic use, kinds of antibiotics used, receipt of carbapenem, receipt of aminoglycosides, endoscopic operation, tracheal intubation, hemofiltration and number of hospitalization days in the ICU. The independent variables were assigned from X_1_ to X_8_. Classification covariates were set to dummy variables, and 2 dummy variables were generated by X2 (X_2(1)_ represented 3 < n < 7, X_2(2)_ represented n ≥ 7).

X_2(1)_ was a high-risk factor for MDR bacterial infection (OR 3.319; 95% CI 1.486–7.414; *P* = 0.003), and X_8_ was a moderate risk factor for MDR bacterial infection (OR 1.048; 95% CI 1.002–1.095; *P* = 0.039) (Table 4).

**Table 4:** Unconditioned logistic regression analysis of risk factors for MDR infection

Risk factors	B	S.E	Wald	df	*P-*value	OR	95% CI
X_2_			9.464	2	0.009		
X_2(1)_	1.200	0.410	8.557	1	0.003	3.319	1.486–7.414
X_2(2)_	− 0.586	0.990	0.350	1	0.554	0.557	0.080–3.874
X_8_	0.047	0.023	4.251	1	0.039	1.048	1.002–1.095

B, partial regression coefficient; S.E, standard error; OR, odds ratio; CI, confidence Interval. X_2_, kinds of antibiotics; X_2(1)_ represented 3 < n < 7, X_2(2)_ represented n ≥ 7; X_8_, hospitalization days in ICU

## Discussion

Our study found that there was no significant difference in the severity of SAP between the MDR and non-MDR groups. The mortality rate in the MDR group was significantly higher than that in the non-MDR group, which indicated that MDR bacterial infection was an important cause of death in SAP patients. This is because as the disease progresses, compensatory anti-inflammatory response syndrome (CARS) and SIRS compound one other and gradually worsen, resulting in mixed antagonistic response syndrome (MARS) [29]. The advantage of a proinflammatory response over an anti-inflammatory response is gradually reversed, and the patient sustains low levels of inflammation with severe immunosuppression development eventually [30]. SAP changes from an early aseptic chemical inflammation to a secondary multisite MDR bacterial infection; uncontrolled pancreatic and severe systemic infections cause sepsis, infectious bleeding, digestive tract spasms and other complications leading to death [31]. However, such findings must be interpreted cautiously because they are probably correlated with the fact that the peak of death occurred in the first and second stage, which was more frequent among patients who were more severely ill, while the MDR bacterial infection occurred later.

The total hospitalization days did not differ significantly between the two groups, which was related to the abandonment of treatment in some patients. Precautionary antibiotic use, kinds of antibiotics used, receipt of carbapenem, tracheal intubation, hemofiltration and number of hospitalization days in the intensive care unit were significantly higher in the MDR group. This indicates that the above interventions were important causes of MDR bacterial infections. Endoscopic surgery was a safe treatment measure for patients [32]. Unconditional logistic regression showed that ICU hospitalization was a risk factor for MDR bacterial infection [33]. When 4 to 6 different kinds of antibiotics used in patients, the risk of MDR bacterial infection was approximately 3 times that of patients given 1 to 3 antibiotics; therefore, we can draw the conclusion that using a variety of antibiotics increases the risk of MDR bacterial infection [34].

SAP infection was caused by pathogens that passed through the blood and bile duct systems or retrograded through the duodenum and ascended into the main pancreatic duct. At the same time, intestinal pathogens crossed the intestinal barrier and then translocated into the lymphatic system and the parenteral system to cause infection [35]. Although gram-negative bacteria were still dominant, the proportion of gram-positive bacteria increased notably compared with 27.9% and 23.9% reported by Ma [4] and Su [36]. One of the reasons is that drainage or postoperative infections occur after the appearance of pancreatic or anastomotic fistula, leading to the emergence of multiple infection foci. However, the reason for the increase in the number of enterococci in SAP patients remains unclear and may be related to the prophylactic use of antibiotics [37]. Fernanda S. Soares [38] et al. found that prophylactic use of meropenem in SAP-affected mice induced *Enterococcus* colonization of the small intestine and gradually became predominant in the gut, which led to an increase in the number of gram-positive bacteria. A multihospital prospective clinical study showed that the intestinal population of *Enterococcus* was higher and more positively correlated with the serum levels of IL-6 in SAP patients than in MAP patients, suggesting that the increase in enterococci contributes to the severity of this disease [39].

The resistance rates of *Escherichia coli* and *Klebsiella pneumoniae* to quinolones were higher than those of nonfermentative bacteria, but the resistance rates to aminoglycosides were the opposite. Resistance to third generation cephalosporins by Enterobacteriaceae, which represents the major mechanism of antimicrobial resistance among *Escherichia coli* isolates [40]. Of the nonfermentative bacteria, *Pseudomonas aeruginosa* and *Acinetobacter baumannii* gradually exhibited resistance to carbapenems through an active efflux system and decreased permeability of the outer membrane [41, 42]. The drug resistance rates were higher than those of *Escherichia coli* and *Klebsiella pneumoniae*; therefore, it was necessary to combine treatment with β-lactamase inhibitors in the clinic [43]. The resistance rates of *Escherichia coli* and *Klebsiella pneumoniae* to cephalosporins were high, while the rates of *Acinetobacter baumannii* to ceftazidime and cefepime were higher than those of *Pseudomonas aeruginosa*. The rates of the main gram-negative bacteria to aztreonam were also higher, but *Klebsiella pneumoniae* and *Acinetobacter baumannii* were more sensitive to the compound sulfamethoxazole.

The detection rate of resistant *Enterococcus faecium* was higher than that of *Enterococcus faecalis*, and the resistance rates of these two bacteria to penicillin were quite different; additionally, the resistance rates to high concentration gentamicin were all over 50%, which was consistent with previous reports [44]. Therefore, the antibacterial effect was poorer for those pathogens when using aminoglycoside-penicillin or benzyl-penicillin for synergistic effects and screening should be performed for clinical use. The detection rate of resistance in *Staphylococcus epidermidis* was higher than that in *Staphylococcus aureus*; however, the resistance rates of *Staphylococcus aureus* to quinolones and gentamicin were higher than those of *Staphylococcus epidermidis*. This is due to the formation of staphylococcus biofilms as an immune evasion and drug resistance mechanism [45]. *Staphylococcus epidermidis* was more sensitive to tetracycline, which was similar to the resistance of *Staphylococcus aureus* to compound sulfamethoxazole.

The prophylactic use of antibiotics for the prevention of secondary pancreatic infection remains controversial [1, 46], and the relevant guidelines are not recommended for patients with SAP and aseptic necrosis [47]. Although prophylactic use of carbapenem antibiotics may lead to bacterial translocation, according to the characteristics of the spectrum, drug sensitivity and antibiotic characteristics, the most appropriate method of empirical antibiotic treatment is meropenem. A short-term, full dose regimen of broad-spectrum antibacterials, especially carbapenems, in the early stage of SAP can eliminate sensitive pathogens quickly, reduce the dual-infection, and reduce the production of drug-resistant strains caused by bacterial flora disturbance. Subsequent use of antimicrobial agents should be based on the results of drug susceptibility testing of pathogens to ensure an effective antibacterial effect, shorten the course of treatment, reduce the production of drug-resistant strains and reduce the probability of fungal infection [48]. In our study, 53% of patients with MDR bacterial infection had received prophylactic antibiotic treatment, and 38% of patients had not received preventive antibiotic treatment. There was no statistical difference between the use of prophylactic antibiotics and MDR bacterial infection.

Our study reports for the first time that more kinds of antibiotics and ICU hospitalization are associated with the development of MDR bacterial infections. This can help clinicians make a better choice to treat AP patients. However, our research also has some limitations. First, this is a single-center retrospective study with a small sample size and further research with larger sample sizes is needed. Moreover, there may be some deviation in the type and quantity of pathogenic bacteria because of the complexity of clinical treatment. Finally, our study cannot completely rule out the key factors for MDR bacterial infections.

## Conclusions

The bacterial spectrum and drug resistance characteristics of SAP patients provide a certain reference for the empirical use of antibiotics and the regulation of intestinal microecological treatments. Our study found that gram-negative bacteria were the most common pathogens in SAP infection, and the proportion of gram-positive bacteria increased notably. Besides, more kinds of antibiotics and ICU hospitalization are risk factors for MDR bacterial infections.

## Data Availability

The datasets analysed during the current study are available from the corresponding author on reasonable request.

## Abbreviations

SAP: Severe acute pancreatitis
IPN: Infectious pancreatic necrosis
MDR: Multi-drug resistant
SIRS: Systemic inflammatory response syndrome
ARDS: Acute respiratory distress syndrome
MODS: Multiple organ dysfunction syndrome
MRSA: Methicillin-resistant Staphylococcus aureus
VRE: Vancomycin-resistant Enterococcus species
MRCNS: Methicillin-resistant coagulasenegative staphylococci
ORs: Odds ratios
CIs: Confidence intervals
ICU: Intensive care units
XDR: Extensively drug resistant
ESBL: Extended Spectrum Beta-Lactamases
CARS: Compensatory anti-inflammatory response syndrome
MARS: Mixed antagonistic response syndrome
